# Constructing Better Classifier Ensemble Based on Weighted Accuracy and Diversity Measure

**DOI:** 10.1155/2014/961747

**Published:** 2014-01-28

**Authors:** Xiaodong Zeng, Derek F. Wong, Lidia S. Chao

**Affiliations:** NLP^2^CT Lab/Department of Computer and Information Science, University of Macau, Taipa 999078, Macau

## Abstract

A weighted accuracy and diversity (WAD) method is presented, a novel measure used to evaluate the quality of the classifier ensemble, assisting in the ensemble selection task. The proposed measure is motivated by a commonly accepted hypothesis; that is, a robust classifier ensemble should not only be accurate but also different from every other member. In fact, accuracy and diversity are mutual restraint factors; that is, an ensemble with high accuracy may have low diversity, and an overly diverse ensemble may negatively affect accuracy. This study proposes a method to find the balance between accuracy and diversity that enhances the predictive ability of an ensemble for unknown data. The quality assessment for an ensemble is performed such that the final score is achieved by computing the harmonic mean of accuracy and diversity, where two weight parameters are used to balance them. The measure is compared to two representative measures, Kappa-Error and GenDiv, and two threshold measures that consider only accuracy or diversity, with two heuristic search algorithms, genetic algorithm, and forward hill-climbing algorithm, in ensemble selection tasks performed on 15 UCI benchmark datasets. The empirical results demonstrate that the WAD measure is superior to others in most cases.

## 1. Introduction

As distinguished from general individual classification methods, including naïve Bayes [1], decision tree [2], and svm [3], the most important idea behind the ensemble methods [4] is the use of a set of base classifiers and combining their predictive capabilities into a single classification task. Through the combination of multiple base classifiers, a more accurate and stronger prediction can be obtained. Ensemble methods can also be understood by comparison to the scenario of people making decisions because people often consider diverse opinions to reach their final decision, thus reducing the risk of making mistakes. In recent decades, many researchers have investigated ensemble technology, resulting in a number of outstanding algorithms proposed in the literature, such as bagging [5], adaboost [6], mixture-of-experts [7], and random forest [8]. Nevertheless, there are two primary shortcomings in generic ensemble methods: efficiency and redundant classifiers. According to the survey results reported by Tsoumakas et al. [9], a large-scale ensemble learning task can easily create thousands of base classifiers, or even more. There is no doubt that having such a large number of classifiers in an ensemble requires large memory and computational overhead. This in turn leads to an increase in the training cost, storage demands, and prediction time. In addition, an ensemble with a large number of classifiers does not always generate better prediction results. This is because an ensemble tends to contain redundant classifiers in addition to high-quality ones. The former negatively affects the overall ensemble predictive performance.

Ensemble selection (i.e., ensemble pruning, ensemble thinning, or classifier selection) is regarded as a type of effective technique to solve these two shortcomings. The goal in ensemble selection is to reduce the memory requirement and accelerate the classification process while preserving or improving the predictive ability [10]. Just as the name implies, ensemble selection refers to the approaches that address the selection of a subset of optimal classifiers from the original ensemble prior to prediction combination. Given an original ensemble with *m* base classifiers *E* = {*C*_1_, *C*_2_,…, *C*_*m*_} and a validation (evaluation, pruning, or selection) dataset with *k* samples *D* = {(*x*_1_, *y*_1_), (*x*_2_, *y*_2_),…, (*x*_*k*_, *y*_*k*_)}, the objective is to form an optimal subensemble *E*′ = {*C*_1_, *C*_2_,…, *C*_*n*_}, where the size of the optimal subensemble, *n*, is less than or equal to the size of the original ensemble, *m* (*n* ≤ *m*). The ensemble selection behavior relies on two core elements, that is, evaluation measure and search method. The score calculated by the evaluation measure is the quality assessment used to guide the ensemble selection. The target to evaluate could be an individual classifier or an ensemble, from which two types of measure can be derived, that is, *classifier* and *ensemble based*. The score assessed from a classifier-based measure represents the quality of an individual classifier; the ensemble-based measure evaluates the quality of the whole ensemble. The goal of the search method is to find the classifiers with high quality scores examined by the evaluation measure. Various ensemble selection approaches are examples of combining an evaluation measure and a searching method [9]. For example, in ranking-based ensemble selection approaches [11, 12], classifiers in the ensemble are reordered in descending order based on their quality scores, and the first *n* (user-defined number) top classifiers are used. Intuitively, a ranking-based approach is the combination of a classifier-based measure and a ranking search method. The advantage of these methods is the low searching complexity, that is, *O*(*m*), because it applies a ranking search algorithm. This approach may sometimes work well, but it is theoretically unsound, as illustrated by the classical example mentioned in [10]: “an ensemble of three identical classifiers with 95% accuracy is worse than an ensemble of three classifiers with 67% accuracy and least pairwise correlated error.” Another representative instance of an optimization-based ensemble selection approach is constructed using an ensemble-based measure and an optimization search method [10, 13–15]. It consists of an optimization process of searching for an optimal subensemble in the space of 2^*m*^ − 1 (nonempty case). The evaluation measure in this case should have the capability of evaluating the quality with respect to the whole ensemble. Unlike the ranking-based methods, it needs the help of an optimization searching algorithm (e.g., a genetic algorithm or a hill climbing algorithm) to avoid exhaustive search complexity, that is, *O*(2^*m*^ − 1).

In this study, ensemble-based measures for optimization-based ensemble selection are emphasized. Two characteristics of ensemble measures are used in this method: (a) they assess the quality of an ensemble with multiple classifiers rather than the quality of an individual classifier, and (b) they usually work with heuristic search algorithms to perform the ensemble selection.

The latent question of ensemble-based evaluation measures is thus “*what is a good classifier ensemble?*” Many researchers have tried to answer this question in their ensemble selection tasks. One comprehensive strategy is to emphasize the ensemble accuracy, so the subensemble with high accuracy stays in the validation dataset. Margineantu and Dietterich [13] first claimed the feasibility of using ensemble accuracy for the ensemble selection task. Zhou et al. proposed the GASEN [14] and GASEN-b [15] selective ensemble learning algorithms, in which the ensemble selection procedures apply genetic algorithm (GA) to search the optimal subensemble according to the majority voting accuracy (MVA) in the validation dataset. In the ensemble selection experiments conducted by Fan et al. [16], two accuracy-class evaluation measures were used, that is, average accuracy and mean squared error. Caruana et al. [17] performed a similar trial, experimenting with several evaluation measures, including root mean squared error, precision/recall *F*-measure, and average precision. Although the experimental results from the above studies illustrated that the selected subensembles based on accuracy measures may provide some improvements with respect to the original ensemble, there exists NO solid proof for the strong correlation between the ensemble accuracy on the validation data and the predictive performance on the test data. However, several studies have proved that too high accuracy may lead to the overfitting problem.

Other scholars insist that the ensemble constructed by a set of diverse classifiers should survive. Such scholars prefer using diversity to represent the ensemble quality. Ruta and Gabrys [18] applied twelve widely known diversity measures, including the disagreement measure, entropy measure, and interrater agreement, in their experiments, to achieve better results than accuracy measures. Martínez-Muñoz and Suárez [12], Banfield et al. [19], and Partalas et al. [20] proposed four similar diversity measures, that is, concurrency, margin distance minimization, complementariness, and the uncertainty aware measure, for selecting subensembles through the greedy search algorithm, producing impressive results. Their results show that computing the degrees of diversity may be a good choice for the evaluation measure. Nevertheless, using diversity as the direct measure of ensemble quality is still a controversial issue. On the one hand, the above studies show promising predictive performance using diversity as the evaluation measure. On the other hand, the theoretical and experimental investigations from Tang et al. [21] concluded that diversity could not be explicitly used for constructing the ensemble, based on directed hill-climbing methods.

From the analysis above, it is determined that it is insufficient to use either accuracy or diversity to represent the ensemble quality. A well-known hypothesis in the ensemble learning community claims that an ensemble with high performance and generalization ability should be simultaneously accurate and diverse [4, 22]. Hence, ensemble selection approaches should endeavor to generate such an ensemble. In other words, the evaluation measures need to assess ensemble quality by considering both accuracy and diversity. However, this is not an easy task because accuracy and diversity are mutual restraint factors, where the ensemble with high accuracy may reduce the diversity and diverse ensembles often will negatively affect accuracy. A number of classifier-based evaluation measures motivated by this idea were proposed in recent decades. In the most representative study in [11], the authors proposed a measure to evaluate each individual classifier's contribution to the whole ensemble by integrating the accuracy and diversity. However, for ensemble-based evaluation measures, there are seldom explorations on assessing the quality by considering both accuracy and diversity.

Therefore, in this study, a new ensemble-based evaluation measure, the weighted accuracy and diversity (WAD) measure, is designed to meet this challenge. The proposed measure has three main features. (1) It is designed to evaluate an ensemble quality and work with heuristic search algorithms to conduct optimization-based ensemble selection. (2) It assesses the ensemble quality by considering both accuracy and diversity. To be more precise, inspired from the *F*-measure [7] in information retrieval, the WAD measure combines accuracy and diversity by obtaining the harmonic mean of both measurements. Two weight factors are appended that contribute to the trade-off between accuracy and diversity. (3) It can automatically trade-off accuracy and diversity because these two weight parameters are learned by a linear programming approach. Empirical results on 15 UCI datasets showed that ensemble selection via the WAD measure produces significantly better results.

The structure of this paper is as follows.  Section 2 introduces the design of the new evaluation measure in detail.  Section 3 reports the experimental tests of the proposed measure, including the corresponding process and final results. The conclusions and discussion are summarized in Section 4.

## 2. Method Design

The primary objective of this work is the design of a novel ensemble-based measure to assess the ensemble quality of the ensemble selection task. As mentioned in Section 1, it is important to consider both accuracy and diversity when assessing ensemble quality. To accomplish this, the method integrates accuracy and diversity measurements in a composite score, formulating a mathematical function *Q* = *f*(Acc, Div), where *Q*, Acc, and Div denote ensemble quality, accuracy and diversity, respectively. Three main obstacles remain to be solved in the design of the measure. (a) The method of calculating accuracy and diversity must be determined. The new measure is expected to integrate accuracy and diversity and, though a number of approaches exist that can calculate both terms, the definitions must be clear in preparation for the subsequent design.  Section 2.1 gives the corresponding descriptions. (b) The form of the new measure must be determined; that is, the function *Q* = *f*(Acc, Div) must be defined. Although several studies have tried to find the solution to this question, there has been no approach to date that has yielded a reasonable composite form using both accuracy and diversity. The new measure tackles this problem by proposing the harmonic mean form to combine accuracy and diversity, as reported in Section 2.2. (c) The method used to balance accuracy and diversity must be determined. In the form of the new measure, two weight parameters are used to balance accuracy and diversity. Weight parameters control the importance of accuracy and diversity. The trade-off process is equivalent to a weight value assignment. Particularly, the weight should be adjusted to the specific dataset. The new measure therefore employs a linear programming technique to automatically estimate the weight value, as described in Section 2.2.

### 2.1. Notations and Definitions

The common notations and definitions summarized in the following are used in the remainder of the paper. Let *E* = {*C*_1_, *C*_2_,…, *C*_*m*_} be an original ensemble containing *m* base trained classifiers, where the classifiers are either homogenous, that is, trained by the same base classification algorithm, or heterogeneous, that is, trained by different classification algorithms. Given a validation dataset with *k* samples, *D* = {*S*_1_, *S*_2_,…, *S*_*k*_}, where *S*_*i*_ = {(*x*_*i*_, *y*_*i*_) | *i* ∈ [1, *k*]} denotes both the *i*th multidimensional input feature vector *x*_*i*_ and the label of the *i*th sample *y*_*i*_ ∈ {*v*_1_, *v*_2_,…, *v*_*l*_}. Denote *h*_*i*_(*x*_*j*_) as the prediction from the *i*th classifier *C*_*i*_ in the ensemble on the *j*th sample *x*_*j*_ and *H*(*x*_*j*_) as the prediction of the original ensemble, *E*, for *x*_*j*_. The prediction collection of all classifiers in *E* on the entire dataset, *D*, is represented by Preds = {*h*_*i*_(*x*_*j*_) | 0 < *j* < *k*; 0 < *i* < *m*}.

It is a straightforward concept that accuracy refers to the correct rate. In the example of an individual classifier, the accuracy on a certain dataset equals the quantity of correct predictions over the total number of samples of the dataset. For an ensemble, however, because the prediction is a collective decision from a set of classifiers, there are various types of accuracy, such as majority voting accuracy, average voting accuracy, and weighted majority voting accuracy. In this work, the most common approach is used, that is, the simple majority (plurality) voting accuracy, to assess the ensemble accuracy. The majority voting accuracy, summarized in Notation 2, is attractive for and adaptable to this task because it only needs to validate and collect statistics for the predictions that are chosen by the majority of the classifiers. Moreover, as one of the simplest and most intuitive ensemble fusion techniques, the majority voting technique is widely used among various ensemble methods, such as bagging [5] and random forest [8].

**Notation 1 (correct/incorrect (1/0) output)**

This type of representation for prediction is well known as an oracle output that only considers the correctness of the solution. Oracle output is used in this study because “it incorporates no a priori knowledge of the data and makes no assumption on what the base classifier is” [21]. Hence, the oracle output provides a general model for the following computation of accuracy and diversity. The oracle output, *o*_*i*_(*x*_*j*_), from the *i*th classifier on *j*th sample, as shown in (1), will have an output of 1 if the training sample *x*_*j*_ is classified correctly by the base classifier *C*_*i*_; otherwise the output is 0, expressed as follows:

(1) $$\begin{array}{c} o_{i}\left(x_{j}\right)=\{\begin{array}{cc} 1, & \text{if}\;h_{i}\left(x_{j}\right)=y_{j},\\ 0, & \text{otherwise}. \end{array} \end{array}$$

**Notation 2 (ensemble accuracy)**

Given an ensemble *E* and a dataset *D* with *k* samples, denote *l* as the number of classifiers from *E* that correctly recognize *x*_*i*_. The oracle output *O*(*x*_*i*_) for the ensemble *E* using the simple majority voting for the input sample, *x*_*i*_, can be expressed by (2) as follows:

(2) $$\begin{array}{c} O\left(x_{j}\right)=\{\begin{array}{cc} 1, & \text{if}\;l>m-l,\\ 1\;\text{or}\;0, & \text{else}\;\text{if}\;l=m-l,\\ 0, & \text{otherwise}, \end{array} \end{array}$$

where *O*(*x*_*i*_)∈{0,1}; that is, 1 denotes that the ensemble prediction is correct if the number of correct predictions, *l*, is greater than the number of incorrect predictions, *m* − *l*, and 0 denotes the case when it is not true. When the number of correct predictions is equal to the number of incorrect predictions, the result is a random selection between 0 and 1. Based on the ensemble oracle output, the ensemble accuracy, acc, for the entire dataset, *D*, can be determined using (3) as follows:

(3) $$\begin{array}{c} \text{Acc}=\frac{\sum_{i=1}^{k}O\left(x_{i}\right)}{k}, \end{array}$$

where the final result is the sum of the ensemble oracle outputs for all samples in the dataset over the size of the dataset. The variable acc varies between 0 and 1, where the higher score means that the ensemble is more accurate.

In the research field of ensemble learning, it is well known that the base classifiers in the ensemble should be as diverse as possible [4, 13, 23]. If a classifier output in the ensemble makes errors, it would be an advantage to have additional output from other, different ensemble members. It is meaningless to combine a set of duplicate classifiers. Diversity measures the difference in the classifiers. Researchers have proposed a variety of diversity measures, such as the Kohavi-Wolpert variance [24], generalized diversity [24], and double-fault measure [25]. In this study, the disagreement measure is used, as illustrated in Notations 3 and 4, as proposed by Skalak [26]. This measure was selected because it is a widely accepted measure to evaluate the diversity, and it has been applied to many ensemble problems. For example, Ho [27] used it to assess the diversity in a decision forest problem, and Lu et al. [11] used it as the diversity measure to calculate the classifier contribution in their study.

**Notation 3 (confusion matrix for two classifiers)**

Assume two classifiers, *C*_*i*_ and *C*_*j*_, and their oracle predictions on *D*, *o*_*i*_(*x*_*k*_).  Table 1 shows a 2 × 2 confusion matrix that records the statistics of four scenarios between two classifiers, where *N* ^01^ represents the number of cases in which the sample is incorrectly predicted by *C*_*i*_ but correctly predicted by *C*_*j*_, *N* ^10^ is the number of cases correctly predicted by *C*_*i*_ but incorrectly predicted by *C*_*j*_, and *N* ^00^ and *N* ^11^ are the number of cases in which the sample is incorrectly predicted by both *C*_*i*_ and *C*_*j*_ and correctly predicted by both *C*_*i*_ and *C*_*j*_, respectively.

**Notation 4 (ensemble diversity)**

Given a dataset *D* with *k* samples, based on the confusion matrix for two classifiers as defined in Notation 3, div⁡_*i*,*j*_ denotes the diversity of the pair of classifiers *C*_*i*_ and *C*_*j*_. The diversity of two classifiers is defined based on the intuition that two diverse classifiers disagree with each other or perform differently on the same data. The diversity therefore is the ratio between the number of cases of disagreement (*N* ^10^ and *N* ^01^) and the total number of all cases (*N* ^00^, *N* ^11^, *N* ^10^, and *N* ^01^) as follows:

(4) $$\begin{array}{c} {\mathrm{div}}_{i,j}=\frac{N^{10}+N^{01}}{N^{00}+N^{11}+N^{10}+N^{01}}. \end{array}$$

Extending (4) to the entire ensemble, *E*, with size *m*, Div denotes the ensemble diversity, which is div⁡_*i*,*j*_, averaged over all pairs of classifiers using (5) as follows:

(5) $$\begin{array}{c} \text{Div}=\frac{2}{m\left(m-1\right)}\sum_{i=1}^{m-1}\;\sum_{j=i+1}^{m}{\mathrm{div}}_{i,j}. \end{array}$$

Because for any pair of classifiers in ensemble *E*, *N* ^00^ + *N* ^11^ + *N* ^10^ + *N* ^01^ = *k*, (5) can be further reduced as follows:

(6) $$\begin{array}{c} \text{Div}=\frac{2}{k\cdot m\left(m-1\right)}\sum_{i=1}^{m-1}\;\sum_{j=i+1}^{m}\left(N_{i,j}^{10}+N_{i,j}^{01}\right). \end{array}$$

Div varies between 0 and 1, where 0 indicates no difference and 1 indicates the highest possible diversity.

### 2.2. Weighted Accuracy and Diversity Measure

As mentioned in Section 1, many studies have shown that ensemble quality is strongly correlated with accuracy and diversity. Additionally, the accuracy and diversity are not directly proportional to the ensemble quality. Too high accuracy may lead to the problem of overfitting; that is, the accuracy of the validation dataset is increased, but worse predictions are achieved on unseen data [28]. However, an ensemble that is too diverse tends to comprise multifarious base classifiers that may seriously reduce the overall ensemble performance [21]. In addition, accuracy and diversity are mutual restraint factors, where classifiers with high accuracy put together may downgrade the complementarity (diversity) and a highly diverse ensemble negatively affects accuracy. There is thus a balance to be achieved between accuracy and diversity that enhances the predictive ability of an ensemble for unknown data. To obtain this balance, accuracy and diversity measurements are integrated, forming a composite form between accuracy and diversity. In other words, if the results of accuracy and diversity for an ensemble have been evaluated, this evaluation can determine if a certain combinational way to generate a more rational score based on those two results can be applied. Inspired by the well-known evaluation method in information retrieval, that is, the *F*-measure or *F*-score [7], considering both the precision and the recall, the WAD ensemble evaluation measure is developed. WAD is an acronym for weighted accuracy and diversity and performs the evaluation score of the ensemble quality by computing the harmonic mean of the accuracy and diversity measurements. According to Sasaki [29], the harmonic mean can create a more reasonable score to balance two factors and is more intuitive than the arithmetic mean when computing a mean of ratios. Particularly, different from the form of the *F*-measure, two parameters are assigned, *α* and *β*, representing the weight of accuracy and diversity, respectively, balance the effects of two factors. The composite form of measuring ensemble quality is therefore defined in Lemma 1 as follows.

**Lemma 1**

Given an ensemble *E* and a dataset *D*, let each classifier in *E* predict all samples in *D*, collecting their results by *Preds*. The ensemble accuracy *Acc* and the ensemble diversity *Div* can be computed according to Notations 2 and 4, respectively. Denote the ensemble quality score as *WAD* and the form by (7) as follows:

(7) $$\begin{array}{c} {WAD}_{\alpha ,\beta }\left(Acc,Div\right)=\frac{Acc\cdot Div}{\beta \cdot Acc+\alpha \cdot Div}, \end{array}$$

where *α* and *β* are two weight parameters that control the importance of accuracy and diversity, respectively. The sum of the two weight parameters equals 1. If the measure focuses more on accuracy, the value of *α* should be greater than *β*. If the measure focuses more on diversity, *α* should be less than *β*.

Given two weights, *α* and *β*, associated with the accuracy and diversity measurements, Acc and Div, respectively, the weighted harmonic mean (WHM) is defined by (8) as follows:

(8) WHM(Acc,Div)=∑ω∈{α,β}ω∑ω∈{α,β};x∈{Acc,Div}(ω/x)=α+βα/Acc+β/Div=Acc·Divβ·Acc+α·Div.

The formula derived in (8) is exactly the same as the form of the WAD measure. The ensemble quality increases with the increasing value of WAD score. The WAD score varies between 0 and 1, where it reaches its best value at 1 and worst value at 0.

When calculating the WAD measure, the ensemble accuracy Acc and ensemble diversity Div can be computed using Notations 2 and 4. However, for the two weight parameters, *α* and *β*, a solution must be proposed to determine their adaptable values. In the rest of this subsection, the estimation of the weight parameters will be discussed. The WAD measure employs the two parameters to balance accuracy and diversity. A straightforward approach is to manually preset the values. However, such a hard-coded approach is irrational and lacks theoretical support because the ensembles are applied to different datasets and should therefore have specific optimal weight values. Ideally, the values should be adjusted to the dataset and could be automatically estimated from the data. In fact, the weight parameter estimation for WAD can be formulated to a constrained linear programming problem, as described by Lemma 2.

**Lemma 2**

Assume the current ensemble *E* with *m* classifiers and the predictions *Preds* of each classifier in *E* on the validation dataset *D*. The ensemble accuracy *Acc* has been computed using Notation 2, and the diversity *Div* has been computed using Notation 4. The estimation of the weight parameters (*α* and *β*) can then be formulated as a linear programming problem, and the corresponding mathematical programming formulation is as follows:

(9) max⁡α,β⁡ (Acc·Div)β·Acc+α·Divs.t. α+β=1if  Acc>Div, αβ≤AccDivelse, αβ≥AccDiv0≤α, β≤1.

The objective function of this problem is expressed by maximizing the WAD score, where *Acc* and *Div* in this case are two constants. Meanwhile the objective function is subject to three constraints, that is, the equality *α* + *β* = 1, and the inequalities if *Acc* > *Div*, *α*/*β* ≤ *Acc*/*Div*, else, *α*/*β* ≥ *Acc*/*Div* and 0 ≤ *α*, *β* ≤ 1, that specify a convex polytope to be optimized. The second constraint is defined according to the intuition that the results of accuracy and diversity are simultaneously required to be as large as possible. If the accuracy result is greater than the diversity result, let the ratio between *α* and *β* be less than or equal to the ratio between accuracy and diversity. If the accuracy result is less than the diversity result, then the ratio between *α* and *β* should be greater than or equal to the ratio between accuracy and diversity.

The function in (9) is a very typical linear programming problem. We can optimize it using the simplex algorithm in [30], developed by Dantzig in 1947, which solves the problem by forming a feasible solution at a vertex of the polytope and then walking along a path on the edges of the polytope to vertices with nondecreasing values of the objective function until an optimum is reached.

The pseudocode of the WAD measure is presented in Pseudocode 1. For computing the WAD score of an ensemble, an original ensemble *E* with *m* base classifiers and a validation dataset *D* with *k* instances should be provided. The computation starts from collecting predictions *h*_*i*_(*x*_*j*_) of each classifier *C*_*i*_ in the ensemble *E* on each data sample *S*_*j*_ in validation dataset *D*. The results are recorded in Preds. The accuracy Acc of the ensemble *E* is then computed based on the prediction results Preds and the approach in Notation 2. Similarly, the diversity Div of the ensemble *E* is computed according to Notation 4 in Preds. Afterwards, the linear programming algorithm is used to estimate the weight parameter values *α* and *β*. In the last step, the WAD score is calculated using (7).

## 3. Evaluation

In this section, the effectiveness of the WAD measure is investigated in ensemble selection tasks. Coupled with two existing representative ensemble evaluation measures and two threshold measures, the proposed measure was combined with two heuristic search algorithms for conducting ensemble selection on 15 UCI benchmark datasets. In the following subsections, the setting of the experiments is introduced, and the results of the comparison experiments are reported.

### 3.1. Experimental Settings

The experimental datasets are taken from the UCI machine learning repository [31]. In the experiments, 15 different datasets are chosen for the evaluation. The characteristics of the various datasets are shown in Table 2. To avoid bias, the datasets are selected as follows: (a) four small-size datasets with less than 500 instances, that is, *hepatitis*, *autos*, *heart*-*statlog*, and *ionosphere*; (b) six medium-size datasets with 500–3,000 instances, that is, *credit*, *diabetes*, *vehicle*, *car*, *cmc*, and *segment*; (c) five large-size datasets with more than 2,000 instances, that is, *kr*-*vs*-*kp*, *hypothyroid*, *waveform-5000*, *page*-*blocks*, and *nursery*. In addition, the experimental datasets cover six binary-class problems and nine multiclass problems. All datasets have removed the samples with missing values. The experimental workbench is WEKA [32], a popular suite of machine learning software written in Java, developed at the University of Waikato.

Initially, each dataset is divided into three disjunctive parts, that is, the *training set*, *validating set*, and *testing set*, each containing 40%, 40%, and 20% of the samples, respectively. The training set is for original ensemble production, the validatingset is for ensemble selection and the testing set is for selected ensemble evaluation. The proportionate stratified sampling is employed to guarantee the balance of class distribution in the three divided sets. Based on the training set, the original ensemble is produced with 200 base classifiers generated using the bagging method [5], where 200 diverse datasets are randomly generated by drawing with replacement amongst *N*, where *N* is the size of the original training set, and then trained up the corresponding 200 base classifiers by the unpruned J48 decision tree, a variant of C4.5 [2].

For the comparison, two existing representative ensemble evaluation measures are used, that is, the Kappa-Error Convex Hull Pruning measure [13] and the GenDiv [33] measure, because their objective is similar to the WAD measure objective. The former is a typical evaluation measure for ensemble selection, considering accuracy and diversity. Several studies [10, 33] employed this method as an important comparison candidate. The latter is the latest representative measure that trades off accuracy and diversity. In addition to those two candidates, two additional threshold measures are used, Acc-Only and Div-Only. The first one takes only the accuracy into consideration, and the quality score is computed according to Notation 2. The second one only assesses the quality score by the diversity, according to Notation 4.

All five candidates of evaluation measure are compared using two common heuristic search algorithms, that is, the genetic algorithm and the forward hill-climbing algorithm, to conduct ensemble selection on the validating set. The genetic algorithm, inspired by evolution and developed by John Holland [1] at the University of Michigan in the 1970s, can be used to yield useful solutions to optimization and search problems. To use a genetic algorithm, the solution for a specific problem should be projected to a genome or chromosome. The genetic algorithm randomly generates a population of chromosomes and utilizes genetic operators such as mutation and crossover operators to evolve the population, producing more diverse chromosomes to find the best one. This search approach has been applied in many ensemble selection tasks [14, 15, 33]. The forward hill-climbing algorithm [34] belongs to a greedy search class of algorithms that focuses on adding or removing a specific classifier such that the improvement in the ensemble performance is maximal. The searching starts from a single best classifier and seeks a pair of classifiers that maximally increases the ensemble performance at each round. As one of the most effective search algorithms, it is also widely used in ensemble selection tasks [13, 16, 17, 35]. In this experiment, the evaluation measures are considered the objective or evaluation function in the search algorithms. The parameters of the search algorithms are set as follows.

- GA: the population size is 50, the crossover rate is 0.8, the mutation rate is 0.7, and the termination condition is no improvement for 100 iterations.
- FHC: the direction is forward, and the termination condition is that it stops at convergence.

Simple majority voting is used to combine the predictions of the selected ensemble on the test set. The size of the resulting ensemble and its classification correct rate of the test data using the combination method are recorded. The whole experiment is performed 10 times for each dataset, and the results are averaged.

### 3.2. Ensemble Size Evaluation

Tables 3 and 4 show the average size of the ensemble selected by all five evaluation measures, that is, WAD, Kappa-Error, GenDiv, Acc-Only and Div-Only, equipped with the two search algorithms, GA and FHC,for the 15 UCI datasets. The last column (No-Selection) of the table lists the size of the original ensemble, and the bottom row reports the average size across all datasets for each measure. The results show that the average size of ensemble selected via the WAD measure ranks in the exact middle of the pack. In the GA search case, the greatest reduction with respect to the original ensemble occurs for Acc-Only, where the average ensemble size is 10.6. The WAD case is in third place, where the size is 24.3, showing a reduction of approximately 12% from the original ensemble. A similar scenario occurs in the FHC search case, where the average ensemble size of WAD is 23.3, also showing an approximate reduction of 12%. The results of the selected ensemble size are shown to testify that, for a selected classifier in the ensemble, *sufficient classifiers are more essential than less ones in an ensemble*. According to a previous experiment [35], the selected ensemble size and predictive performance are not strongly correlated. Although ensemble selections via other measures such as Kappa-Error and Acc-Only exhibit a greater reduction in ensemble size, there are fewer than five classifiers left in several cases, indicating that such a situation may be unreasonable and unreliable. Breiman [5] and Opitz and Maclin [36] proposed that in most ensemble cases, most or all of the generalization can be gained in a well-constructed ensemble with 25 base classifiers. The results in Tables 3 and 4 demonstrate that the WAD results (24.3 and 23.3) fit this golden size. In addition to the ensemble size, good quality is a better target for the classifier. The following experimental results validate that the ensemble selection via the WAD measure can generate the ensemble not only with reasonable size but also with robust performance.

### 3.3. Ensemble Quality Evaluation

Tables 5 and 6 summarize the predictive performance for 15 datasets with all five candidate evaluation measures, that is, WAD, Kappa-Error, GenDiv, Acc-Only, and Div-Only.  Table 5 reports the classification correct rate with ensemble selection using the GA search method, and Table 6 reports the classification correct rate using the FHC search method. The last column, No-Selection, in both tables, indicates the performance of the original ensemble without any ensemble selection process. Each cell in these two tables records the mean and standard deviation value of the 10 runs of the experiment. The bottom row illustrates the win/loss/tie summary that is computed using a pairwise *t*-test at 95% significance level. To comprehensively probe the proposed measure, three comparisons are made based on the empirical results in Tables 5 and 6. (1) WAD versus No-Selection. The ensemble selected via the WAD measure outperforms the original ensemble in the overwhelming majority of cases, where WAD + GA achieved 13 significant wins and WAD + FHC achieved 12 significant wins among 15 datasets. Furthermore, there is not a single case of significant loss. Although the WAD on three datasets, that is, car, segment and kr-vs-kp, does not win significantly, it is still comparable to No-Selection. This comparison reveals that ensemble selection using the WAD measure can dramatically upgrade the predictive performance compared to original ensembles. It further shows that fewer classifiers can be employed to preserve or even improve predictive ability. (2) WAD versus Acc-Only and Div-Only, because the goal of the WAD measure is to balance accuracy and diversity, the comparison with the two threshold cases that consider either accuracy or diversity enables the direct demonstration of the performance of the WAD measure with respect to them. To date, no sufficient evidence has been published to support that Acc-Only or Div-Only outperforms No-Selection. Both threshold methods produced poorer results than No-Selection over seven datasets in Tables 5 and 6. This result verified the commonly accepted hypothesis that to consider *only *accuracy or diversity in ensemble selection is inadequate for producing good classifiers and may degrade the predictive performance. The WAD predictions are superior to Acc-Only and Div-Only in most of the cases. As shown in Tables 5 and 6, there is only one (6%) significant loss, and the average rate of significant wins is approximately 75%. In particular, for the significant loss cases (20 in total) when comparing Acc-Only (9 cases) or Div-Only (11 cases) against No-Selection, WAD is still able to come out ahead. This result shows that taking both accuracy and diversity into consideration helps improve the quality of the ensemble selection task. (3) WAD versus Kappa-Error and GenDiv, this comparison is performed between WAD and two state-of-the-art evaluation measures, that is, Kappa-Error and GenDiv. WAD outperforms Kappa-Error and GenDiv on ten and nine datasets out of fifteen cases under both search methods. The results in Tables 5 and 6 also show that the maximum number of significant losses is only two, made with GenDiv.

In summary, under the same search algorithm, the performance of ensemble selection relies strongly on the evaluation measure. The experimental results clearly demonstrate that WAD outperforms other evaluation measures by simultaneously considering both accuracy and diversity, as well as balancing their influence in assessing the ensemble quality. The measure is capable of computing a rational score to guide good ensemble selection. The comparison results with Acc-Only and Div-Only strongly support this. Furthermore, unlike Kappa-Error and GenDiv, the balance between accuracy and diversity in WAD is performed in a way that the accuracy and diversity weights are learned automatically from the validating set. The learned parameters therefore can better represent the characteristics of the given datasets and maximally contribute to performance improvement.

### 3.4. Analysis of Four Representative Cases

**Figure 1:**
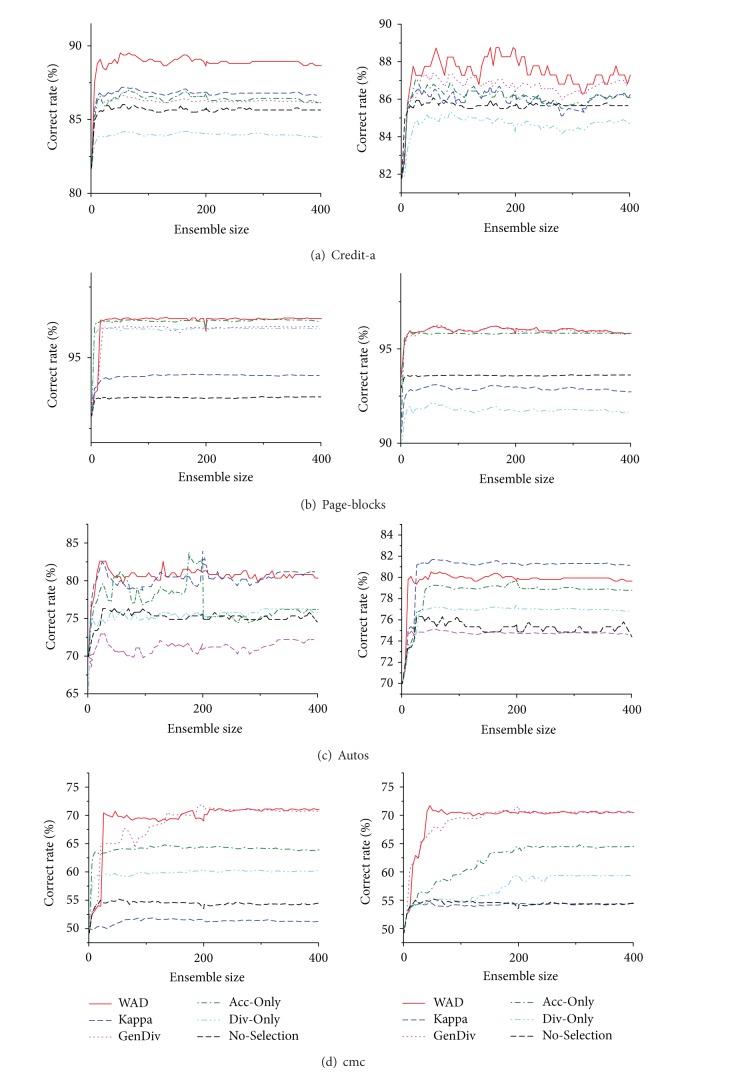
The curves of the classification correct rate on four representative datasets: credit-a, page-blocks, autos, and cmc. The left graphs report the ensemble selection cases using FHC search, while the right ones using GA search.

In this subsection, four representative datasets were extracted according to the empirical results of Tables 5 and 6: (a) credit-a, the case in which WAD outperforms all other approaches; (b) page-blocks, the case in which WAD did not outperform both Acc-Only and Div-Only; (c) autos, the case in which Kappa-Error outperforms WAD; and (d) cmc, the case in which GenDiv outperforms WAD.  Figure 1 shows the curves of average correct rate for these four datasets with respect to the specific original ensemble size. The same experimental settings are used as in the previous experiments, but the size of the original ensemble is increased progressively (the ensemble size ranged from 3 to 400).

The first observed target is focused on the baseline case of No-Selection, in which, with the increase in the ensemble size, the classification correct rate grows placidly until approximately 30 classifiers. The ensemble then begins to overfit with large ensemble sizes (>30), and the improvement appears to become nearly asymptotic to a plateau. This phenomenon is consistent with the claim in the ensemble selection community that combining all of the original ensembles does not always give better performance [9, 11, 14, 20, 35]. The second observed target is shifted to the five ensemble selection cases, that is, WAD, Kappa-Error, GenDiv, Acc-Only, and Div-Only. The ensemble selections with ascending original ensemble size allow an easier verification of the generalization ability of the ensemble selection. Intuitively, a larger ensemble should provide more classifier candidates for constructing a better subensemble. At the same time, however, the chances of picking “bad” classifiers for the subensemble are improved. The delicate ensemble selection measures therefore tend to produce unfavorable results in this situation, and the selected ensemble performs worse than the original ensemble.  Figure 1 shows that the ensemble selection via WAD gave the best performance on each dataset. It not only achieved the advantages of the datasets, as shown in Figure 1(a), where WAD outperformed others in the last experiment, but the ensemble selection also retrieved the situation when the other measures outperformed WAD, as shown in Figures 1(b) and 1(d). This outcome shows that the WAD measure allows the corresponding ensemble selection to achieve high generalization ability. There was only one exceptional case found, in Figure 1(c), when the Kappa-Error performed better than WAD when conducting the ensemble selection with GA search. In reality, it is impossible and unrealistic to request the new measure to be superior to all others under whatever circumstances.

## 4. Conclusion and Future Works

This study introduces a novel and effective evaluation measure, that is, the weighted accuracy and diversity (WAD), for the ensemble selection task. The goal of the proposed measure is to assess the ensemble quality with respect to the whole ensemble. Simultaneously considering and balancing accuracy and diversity are the best solution for the ensemble quality evaluation. To achieve this goal, the proposed measure performs the evaluation in a different way such that the final quality score for an ensemble is a combination of accuracy and diversity measurement. Inspired by the *F*-measure evaluation approach in information retrieval, the ensemble quality score is determined by computing the harmonic mean of accuracy and diversity. Additionally, two weight parameters are assigned to balance accuracy and diversity. Another feature of the proposed measure is that the values of the weight parameter are automatically learned from the data. Experimental comparisons on 15 UCI datasets indicate that ensemble selection via the WAD measure can produce the ensemble with a reasonable size and robust performance and that WAD performs better than three baseline cases, that is, No-Selection, Acc-Only, and Div-Only, and better than two existing measures, that is, Kappa-Error and GenDiv.

Several improvements of the current version of the measure are possible. First, to compute accuracy and diversity, the scope of this study is limited to two specific methods, majority voting accuracy and disagreement diversity. However, the question of employing other accuracy and diversity methods while achieving favorable results can still be answered. Second, the balance between accuracy and diversity is still a controversial issue. In this paper, the problem is resolved using a value assignment for the weights *α* and *β*. Their values are adjusted to the validating set using a linear programming technique. However, advance knowledge of the result of accuracy and diversity is required to apply the technique. An interesting improvement would be to trade off the accuracy and diversity when computing them. Third, in addition to accuracy and diversity, there may be other factors that can be used to help evaluate ensemble quality. If so, what are they, and what is their form? Future works will involve evaluating the current version of the WAD measure in other ensemble selection tasks that work on different datasets, original ensembles, and search algorithms and optimizing the current version to find a better version of the WAD measure by considering these possible improvements.

## Figures and Tables

**Pseudocode 1:**
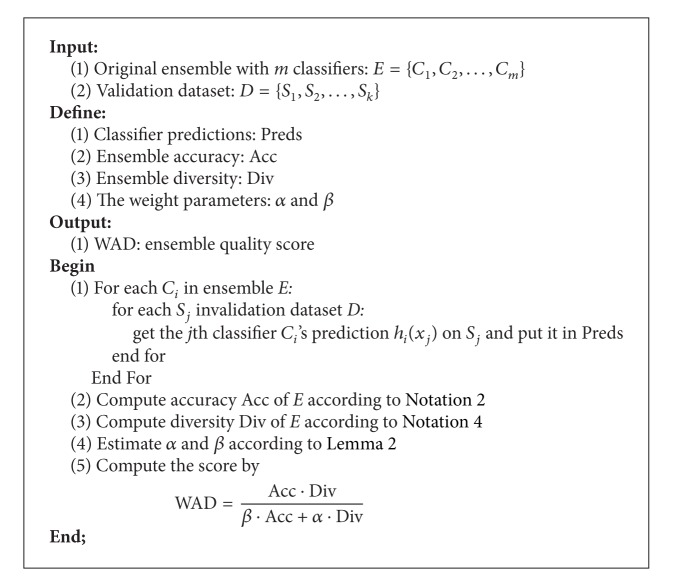
The pseudocode to compute the WAD score.

**Table 1:** Confusion matrix for two classifiers. (*√*) denotes the correct prediction given by the classifier and (×) denotes incorrect prediction.

	*C*_*j*_ (*√*)	*C*_*j*_ (×)
*C*_*i*_ (*√*)	*N* ^11^	*N* ^10^
*C*_*i*_ (×)	*N* ^01^	*N* ^00^

**Table 2:** Experimental datasets from the UCI Repository.

No.	Name	Feature	Instance	Class
1	Hepatitis	19	155	2
2	Autos	26	205	6
3	Heart-statlog	13	270	2
4	Ionosphere	34	350	3
5	Credit	15	690	2
6	Diabetes	8	768	2
7	Vehicle	18	946	4
8	Car	6	1728	4
9	cmc	9	1473	3
10	Segment	19	2310	7
11	kr-vs-kp	36	3196	2
12	Hypothyroid	29	3772	4
13	Waveform-5000	40	4999	3
14	Page-blocks	10	5473	5
15	Nursery	7	12960	5

**Table 3:** The average size of ensembles selected by candidate measures and GA.

Dataset	WAD	Kappa-Error	GenDiv	Acc-Only	Div-Only	No-Selection
Hepatitis	23	11	40	10	25	200
Autos	20	22	34	9	32	200
Heart-statlog	28	13	46	8	34	200
Ionosphere	30	10	34	13	45	200
Credit-a	25	5	34	15	56	200
Diabetes	27	12	34	12	125	200
Vehicle	22	3	27	14	111	200
Car	23	23	32	7	78	200
cmc	26	17	89	6	65	200
Segment	25	19	78	1	34	200
kr-vs-kp	20	16	45	6	43	200
Hypothyroid	29	2	78	18	76	200
Waveform	19	9	10	16	56	200
Page-blocks	24	20	34	11	12	200
Nursery	23	7	65	13	34	200

Average	24.3	12.6	45.3	10.6	55.1	200

**Table 4:** The average size of subensembles selected by candidate measures and FHC.

Dataset	WAD	Kappa-Error	GenDiv	Acc-Only	Div-Only	No-Selection
Hepatitis	27	5	31	9	59	200
Autos	21	12	36	14	43	200
Heart-statlog	20	23	56	11	54	200
Ionosphere	17	4	53	10	65	200
Credit-a	27	3	40	15	34	200
Diabetes	25	9	26	3	66	200
Vehicle	29	18	29	13	56	200
Car	18	11	19	9	69	200
cmc	22	18	43	2	45	200
Segment	24	10	109	4	43	200
kr-vs-kp	25	9	69	9	12	200
Hypothyroid	23	25	33	12	34	200
Waveform	15	10	30	27	78	200
Page-blocks	29	16	37	6	45	200
Nursery	28	7	36	4	78	200

Average	23.3	12	43.1	9.9	52.1	200

**Table 5:** The classification correct rate (%) of subensembles selected by candidate measures and GA using a pairwise *t*-test at a 95% significance level.

Dataset	WAD	Kappa-Error	GenDiv	Acc-Only	Div-Only	No-Selection
Hepatitis	85.31 ± 1.5	81.09 ± 1.65 (5.98)	78.21 ± 1.1 (12.07)	79.2 ± 0.82 (11.3)	86.25 ± 0.97 (−1.67)	80.76 ± 2.14 (5.5)
Autos	79.67 ± 0.92	81.13 ± 0.23 (−4.87)	74.66 ± 0.86 (12.58)	79.86 ± 2.52 (−0.23)	77.48 ± 0.52 (6.55)	75.61 ± 3.25 (3.8)
Heart-statlog	85.5 ± 2.14	83.47 ± 0.5 (2.92)	83.67 ± 1.01 (2.44)	83.25 ± 2.26 (2.28)	79.84 ± 2.28 (5.72)	81.3 ± 0.94 (5.68)
Ionosphere	93.86 ± 1.27	92.07 ± 1.94 (2.44)	95.19 ± 1.42 (−2.21)	93.58 ± 0.88 (0.57)	92.06 ± 2.1 (2.31)	91.72 ± 1.75 (3.12)
Credit-a	88.63 ± 1.29	86.82 ± 0.98 (3.53)	86.37 ± 2.69 (2.39)	86.81 ± 0.6 (4.04)	84.03 ± 0.54 (10.4)	85.62 ± 2.34 (3.56)
Diabetes	83.78 ± 0.87	83.77 ± 0.4 (0.03)	79.75 ± 2.66 (4.55)	75.86 ± 0.78 (21.43)	72.77 ± 2.42 (13.53)	77.54 ± 2.43 (7.64)
Vehicle	78.67 ± 0.69	74.7 ± 1.24 (8.84)	74.65 ± 1.29 (8.68)	70.28 ± 2.31 (11)	71.45 ± 1.01 (18.66)	74.76 ± 1.87 (6.2)
Car	94.81 ± 1.16	92.71 ± 0.71 (4.88)	95.05 ± 0.61 (−0.58)	89.97 ± 0.4 (12.47)	95.18 ± 2.6 (−0.42)	93.7 ± 2.01 (1.51)
cmc	70.34 ± 1.43	54.41 ± 1.24 (26.61)	71.87 ± 0.33 (−3.3)	64 ± 2.09 (7.91)	59.19 ± 2.86 (11.02)	53.31 ± 1.4 (26.91)
Segment	98.58 ± 1.76	97.58 ± 2.79 (0.95)	97.78 ± 0.33 (1.41)	98.67 ± 0.49 (−0.16)	93.77 ± 1.13 (7.27)	97.49 ± 0.46 (1.89)
kr-vs-kp	97.09 ± 0.35	96.07 ± 1.91 (1.66)	96.04 ± 2.39 (1.37)	91.66 ± 2.52 (6.74)	93.13 ± 1.05 (11.31)	95.28 ± 0.65 (7.75)
Hypothyroid	93.22 ± 0.68	90.28 ± 1.35 (6.15)	89.45 ± 1.73 (6.41)	89.84 ± 1.9 (5.29)	88.98 ± 1.65 (7.51)	90.4 ± 0.69 (9.2)
Waveform	86.06 ± 1.42	86.08 ± 0.4 (−0.05)	83.68 ± 0.36 (5.13)	80.42 ± 1.72 (7.99)	84.05 ± 0.28 (4.39)	83.6 ± 1.58 (3.66)
Page-blocks	95.8 ± 1.22	92.92 ± 1.79 (4.2)	96.16 ± 0.21 (−0.92)	95.92 ± 2.51 (−0.14)	91.67 ± 2.4 (4.85)	93.57 ± 1.77 (3.28)
Nursery	95.02 ± 0.99	93.45 ± 2.46 (1.87)	92.56 ± 1.51 (4.3)	93.41 ± 1.73 (2.55)	95.52 ± 1.66 (−0.82)	92.41 ± 1.56 (4.46)

Absolute w/l/t		13/2/0	11/4/0	12/3/0	12/3/0	15/0/0
Significant w/l/t		9/1/5	9/2/4	11/0/4	12/0/3	13/0/2

**Table 6:** The classification correct rate (%) of subensembles selected by candidate measures and forward hill-climbing search algorithm using a pairwise *t*-test at a 95% significance level.

Dataset	WAD	Kappa-Error	GenDiv	Acc-Only	Div-Only	No-Selection
Hepatitis	83.64 ± 1.48	79.7 ± 1.61 (5.69)	76.24 ± 2.1 (9.1)	78.55 ± 2.47 (5.58)	83.82 ± 0.9 (−0.33)	80.76 ± 0.35 (5.98)
Autos	81.1 ± 0.74	83.99 ± 2.75 (−3.21)	71.66 ± 1.77 (15.56)	82.88 ± 2.21 (−2.42)	78.18 ± 0.93 (7.76)	75.61 ± 0.29 (21.84)
Heart-statlog	85.94 ± 2.52	84.72 ± 2.28 (1.13)	82.23 ± 0.71 (4.48)	86.94 ± 2.67 (−0.87)	77.94 ± 1.97 (7.9)	81.3 ± 0.14 (5.81)
Ionosphere	93.61 ± 1.6	93.68 ± 0.12 (−0.14)	93.7 ± 0.58 (−0.17)	90.81 ± 1.18 (4.45)	89.41 ± 1.39 (6.26)	91.72 ± 1.56 (2.67)
Credit-a	91.01 ± 1.07	87.22 ± 0.24 (10.92)	88.96 ± 1.61 (3.35)	87.16 ± 2.09 (5.18)	84.87 ± 0.52 (16.32)	85.62 ± 1.41 (9.62)
Diabetes	83.26 ± 2.64	80.58 ± 1.77 (2.66)	80.42 ± 2.67 (2.39)	75.31 ± 1.56 (8.19)	73.56 ± 2.35 (8.67)	77.54 ± 1.9 (5.56)
Vehicle	81.03 ± 0.31	76.67 ± 0.99 (13.29)	74.12 ± 0.13 (65)	72.45 ± 2.61 (10.32)	71.52 ± 1.84 (16.11)	74.76 ± 2.7 (7.29)
Car	94 ± 0.53	92.16 ± 2.95 (1.94)	93.8 ± 1.22 (0.47)	91.82 ± 2.25 (2.98)	94.97 ± 2.16 (−1.38)	93.7 ± 1.65 (0.54)
cmc	69.02 ± 1.45	51.62 ± 1.98 (22.42)	71.88 ± 0.68 (−5.65)	64.18 ± 2.77 (4.89)	60.48 ± 2.06 (10.72)	53.31 ± 1.58 (23.16)
Segment	97.73 ± 1.67	95.03 ± 2.57 (2.78)	98.98 ± 0.07 (−2.37)	97.26 ± 0.37 (0.86)	91.41 ± 1.37 (9.25)	97.49 ± 1.02 (0.38)
kr-vs-kp	94.99 ± 1.82	96.89 ± 1.28 (−2.71)	94.9 ± 1.86 (0.1)	92.01 ± 2.08 (3.4)	92.52 ± 1.89 (2.97)	95.28 ± 1.78 (−0.37)
Hypothyroid	94.07 ± 0.78	91.04 ± 0.42 (10.81)	89.88 ± 0.81 (11.78)	91.43 ± 2.69 (2.98)	94.72 ± 0.87 (−1.76)	90.4 ± 1.39 (7.28)
Waveform	88.49 ± 1.87	83.23 ± 2.04 (6.01)	81.93 ± 0.47 (10.75)	83.03 ± 2.75 (5.19)	81.27 ± 0.35 (12)	83.6 ± 1.74 (6.05)
Page-blocks	95.93 ± 0.22	90.01 ± 1.11 (16.54)	96.08 ± 0.61 (−0.74)	96.1 ± 0.57 (−0.88)	96.27 ± 0.64 (−1.59)	93.57 ± 2.54 (2.92)
Nursery	94.56 ± 0.26	92.04 ± 2.3 (3.44)	92.81 ± 1.97 (2.78)	90.63 ± 2.82 (4.38)	93.07 ± 0.5 (8.36)	92.41 ± 0.19 (21.11)

Absolute w/l/t		12/3/0	11/4/0	12/3/0	11/4/0	14/1/0
Significant w/l/t		10/2/3	9/2/4	11/1/3	11/0/4	12/0/3
